# Inflammation after spinal cord injury: a review of the critical timeline of signaling cues and cellular infiltration

**DOI:** 10.1186/s12974-021-02337-2

**Published:** 2021-12-07

**Authors:** Daniel J. Hellenbrand, Charles M. Quinn, Zachariah J. Piper, Carolyn N. Morehouse, Jordyn A. Fixel, Amgad S. Hanna

**Affiliations:** Department of Neurological Surgery, School of Medicine and Public Health (UWSMPH), University of Wisconsin, 600 Highland Ave, Madison, WI 53792 USA

**Keywords:** Spinal cord injury, Inflammation, Secondary cascade, Macrophages, Cytokines, Microglia, Astrocytes

## Abstract

Traumatic spinal cord injury (SCI) is a devastating neurological condition that results in a loss of motor and sensory function. Although extensive research to develop treatments for SCI has been performed, to date, none of these treatments have produced a meaningful amount of functional recovery after injury. The primary injury is caused by the initial trauma to the spinal cord and results in ischemia, oxidative damage, edema, and glutamate excitotoxicity. This process initiates a secondary injury cascade, which starts just a few hours post-injury and may continue for more than 6 months, leading to additional cell death and spinal cord damage. Inflammation after SCI is complex and driven by a diverse set of cells and signaling molecules. In this review, we utilize an extensive literature survey to develop the timeline of local immune cell and cytokine behavior after SCI in rodent models. We discuss the precise functional roles of several key cytokines and their effects on a variety of cell types involved in the secondary injury cascade. Furthermore, variations in the inflammatory response between rats and mice are highlighted. Since current SCI treatment options do not successfully initiate functional recovery or axonal regeneration, identifying the specific mechanisms attributed to secondary injury is critical. With a more thorough understanding of the complex SCI pathophysiology, effective therapeutic targets with realistic timelines for intervention may be established to successfully attenuate secondary damage.

## Background

Every year in North America approximately 12,500 people are paralyzed due to a spinal cord injury (SCI) [1]. After SCI, there is a primary injury caused by the initial trauma, which compromises neurons and glia and initiates a secondary injury cascade that leads to additional cell death and spinal cord damage over the subsequent weeks. This overwhelming inflammatory response in the early phase of injury, combined with the disrupted blood–spinal cord barrier, progressively adds to spinal cord swelling and damage. The end result in the chronic stage is a dense glial scar leaving patients with a loss of both sensory and motor function below the level of injury [2]. The paralysis often leaves patients unable to care for themselves, exemplifying the need to develop a treatment to help retain or restore function after SCI.

Inflammation after SCI is complex and orchestrated by many cell types and numerous inflammatory cytokines including tumor necrosis factor alpha (TNFα), interleukin-1β (IL-1β), and interleukin-6 (IL-6), among several others. Although there are several positive effects from inflammation after SCI, the extensive infiltration of immune cells is a principal contributor to neural degeneration [3, 4]. These immune cells are guided to the lesion site from the periphery via cytokines and chemokines released by microglia, astrocytes, and peripherally derived macrophages (PDMs) within the lesion [5, 6]. Overall, there is a general consensus among scientists regarding the inflammation process after SCI. However, there are discrepancies in the inflammation timeline and the extent of cytokine regulation after injury in the literature. Delineation of precise functional roles of cytokines and their timeline of upregulation/downregulation may provide insight into how to regulate acute inflammatory reactions after SCI [7].

The objective of this review is to conduct an extensive survey of the literature to develop a precise timeline of immune cell and cytokine regulation in the spinal cord after SCI. Here we discuss the injury timeline with a focus on cellular behavior, cytokine activity, and the corresponding changes that occur to the injury site. The majority of studies on inflammation after SCI utilize rodent models. After SCI, mice develop fibrous connective tissue domains throughout the injury site, which differs from rats and other mammals that develop necrotic lesion cavities encased by reactive astrocytes [8–12]. Thus, we will also discuss differences in cytokine profiles observed between rats and mice.

## Literature review

**Fig. 1:**
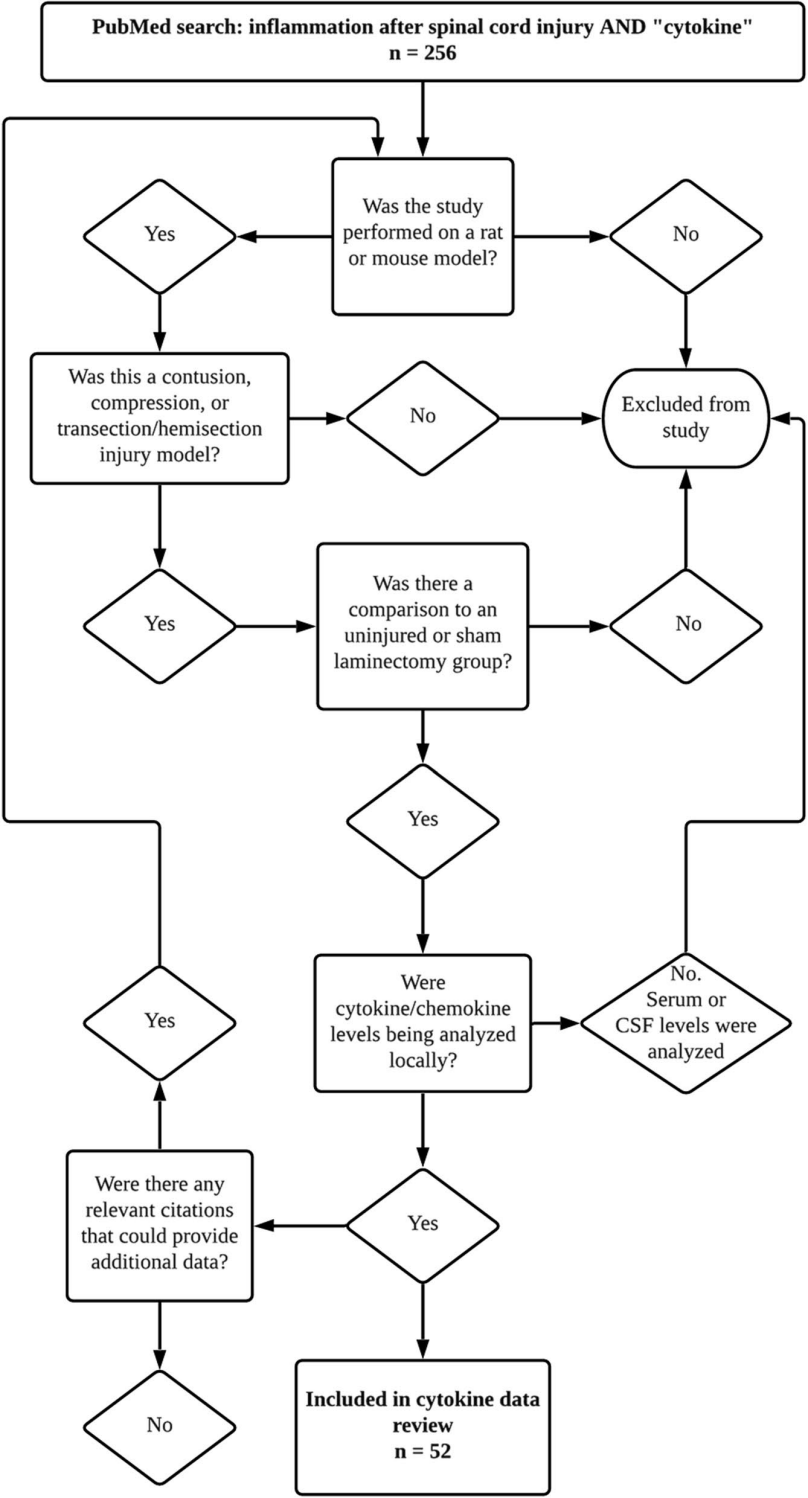
Flowchart displaying how articles were screened to be analyzed for assessing cytokine/chemokine regulation after SCI. All studies used were performed in rats and mice and assessed local cytokine/chemokine regulation at specific times post-injury compared to uninjured or sham controls

An electronic search of the Medline database for literature describing animal models of SCI from 1946 to 2021 was performed using the following conditions: SCI (MeSH Terms) AND inflammation (MeSH Terms) OR secondary injury (MeSH Terms). For the timeline of local cytokine regulation, the results were further screened and only included articles utilizing rats or mice with uninjured controls or sham controls for comparison of local upregulation or downregulation of cytokines within their experiments (Fig. 1).

## Main text

### Inflammation after SCI

**Fig. 2:**
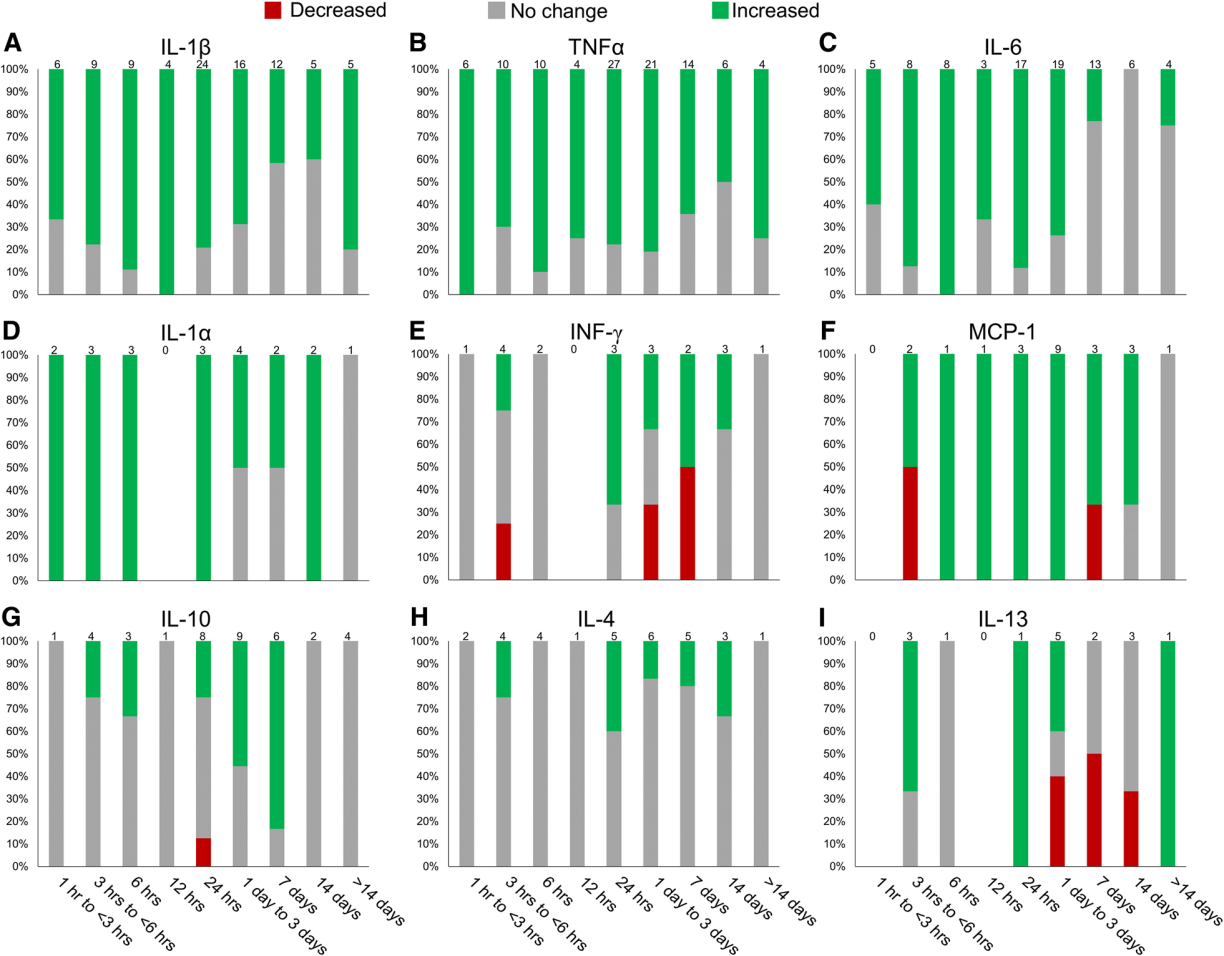
Depiction of cytokine regulation following SCI in rodent models. A literature search was conducted and relevant data regarding significant cytokine regulation was collected at various timepoints. Data is presented as a percentage of studies that found significant changes in cytokine protein or mRNA expression levels compared to sham or naïve controls (*p* < 0.05). The number of papers used for each timepoint is listed at the top of each bar. **A** Changes in IL-1β levels after SCI [17, 21, 37, 38, 40–43, 45–47, 66–68, 85, 87–89, 94, 106, 107, 169–187]. The proinflammatory cytokine IL-1β shows consistent upregulation in the acute phase following SCI. However, there are some discrepancies as to whether IL-1β remains upregulated several days after injury and the second surge 14 days was only observed in mice. **B** Changes in TNFɑ levels after SCI [17, 36–42, 45–48, 66–69, 85, 87–89, 94–96, 107, 121, 169–180, 182–189]. The majority of studies show an upregulation of the proinflammatory cytokine TNFɑ immediately following SCI and persisting several days after injury. **C** Changes in IL-6 levels after SCI [17, 37, 38, 40–42, 45, 47, 66–68, 84, 85, 87–90, 93, 95, 96, 107, 121, 173, 174, 176, 178, 183, 184, 186–189]. Consistent upregulation of the proinflammatory cytokine is seen in the first 24 h following injury before returning to baseline levels by 7-day post-injury. **D** Changes in IL-1ɑ levels after SCI [17, 38, 40, 66, 84, 87, 90, 95]. The proinflammatory cytokine IL-1ɑ is upregulated in a similar manner to its isoform IL-1β, though IL-1β plays a more significant role following SCI [132, 133]. **E** Changes in IFN-γ levels after SCI [17, 38, 48, 87, 90, 95]. The relative change in IFN-γ expression following SCI remains controversial, as shown by the conflicting data presented. It appears to be upregulated in mice and downregulated in rats after 24 h. **F** Changes in MCP-1 levels after SCI [17, 38, 47, 84, 85, 87–90, 93, 121]. While there is some debate surrounding the regulation of the MCP-1 chemokine immediately after injury (1 h to < 6 h), nearly all data collected shows that MCP-1 expression levels elevate quickly and remain upregulated for several days. **G** Changes in IL-10 levels after SCI [17, 21, 38, 46, 47, 66, 85, 87–90, 96, 170, 173–176]. A delayed response is seen with IL-10 showing mixed results until upregulation at 3–7 days after injury. The anti-inflammatory cytokine returns to baseline levels by 14 days. **H** Changes in IL-4 levels after SCI [7, 17, 38, 47, 48, 66, 70, 87]. While some studies show increased expression of the anti-inflammatory IL-4, most researchers did not observe a change in IL-4 levels. **I** Changes in IL-13 levels after SCI [17, 38, 47, 48, 87, 90]. Previous studies display conflicting data surrounding the regulation of IL-13 after injury, where it was upregulated in mice and downregulated in rats 3 days hours post-injury

**Fig. 3:**
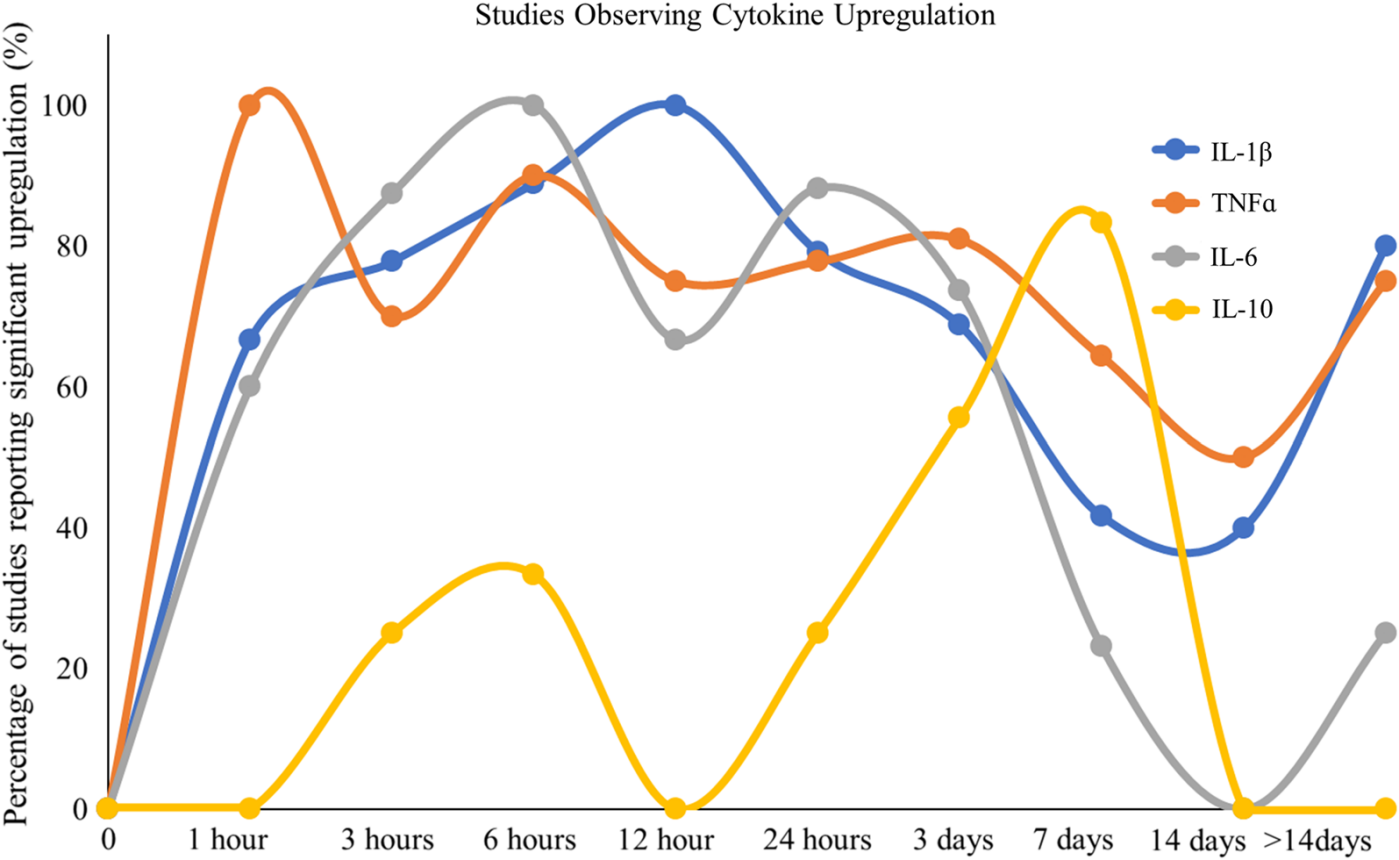
Line graph displaying the percentage of studies that observed significant upregulation in IL-1β, TNFα, IL-6, and IL-10. The majority of investigators observe a significant upregulation of the three most investigated inflammatory cytokines early after injury and TNFα and IL-1β remain upregulated. The anti-inflammatory cytokine IL-10 lags further behind and the majority of investigators show it peaking around 1-week post-injury

Neuroinflammation is the activation of the central nervous system’s (CNS) innate immune system in response to an inflammatory challenge, which is characterized by a host of cellular and molecular changes within the CNS. This inflammation is mediated by the upregulation of cytokines/chemokines (Table 1), which are produced by resident microglia, astrocytes, peripherally derived immune cells, and endothelial cells. Three inflammatory cytokines, TNF, IL-1, and IL-6 have been studied extensively and are upregulated within hours after the initial injury (Table 1, Figs. 2 and 3). This production in inflammatory cytokines and chemokines results in extensive infiltration of immune cells including microglia, PDMs, and neutrophils, which continue the production of additional inflammatory mediators. Although the degree of neuroinflammation depends on the extent of the primary stimulus or insult, the primary insult often results in an overreaction of the inflammatory process after CNS injury leading to additional cell death [13].

**Table 1:** Cytokines and chemokines involved in inflammation after SCI

Names *Aliases*	Cells that secrete the cytokine/chemokine, receptors they bind to, and their effects after SCI
IL-1α *IL-1F1*	Released via activated microglia and PDMs largely in response to disease, infection, or inflammatory events [52] Binds to IL-1R1 [52] Required for neutrophil recruitment during cell death-induced sterile inflammation [128]
IL-1β *IL-1F2*	Produced by activated microglia and PDMs as a proprotein, which is proteolytically processed to its active form by caspase 1 (CASP1/ICE) [52, 129–131] Binds to the IL-1R [129] Plays a more substantial role than IL-1α post-SCI [52, 132, 133] Stimulates inflammatory mediators including prostaglandins, cyclooxygenase 2, and phospholipase A2 [52, 129]
IL-2	Produced by activated T-cells [134] Binds to IL-2R complex, which consists of IL-2Rα, IL-2Rβ, and the common γ-chain subunits [134] Contributes to the proliferation of T-helpers [87] Responsible for initiating the proper response of memory T-cells to invading pathogens [135]
IL-4	Produced by T-helper cells, eosinophils, basophils, and mast cells [136] Binds to IL-4Rɑ, which will either dimerize with the common cytokine-receptor γ-chain and produce the type-1 signaling complex, or with IL-13Rα1 and produce the type-2 signaling complex [136, 53] Increases microglia/macrophages expressing antigens characteristic of an anti-inflammatory M2 phenotype [70] Increases the number of oligodendrocytes and neuronal markers βIII-tubulin and NeuN after SCI, suggesting a role in neuroprotection [137]
IL-5	Produced by hematopoietic and non-hematopoietic cells, including granulocytes, T-cells, and natural helper cells [138] Binds to IL-5R and stimulates B-cell growth as well as increases immunoglobulin secretion (primarily IgA). Is also a key mediator in eosinophil activation [139, 140]
IL-6	Expressed in astrocytes, microglia and PDMs, and neurons [37] Binds to IL-6R, which exists either as a membrane-bound receptor or a soluble receptor [141] Activates inflammation and is a strong recruiter of immune cells after SCI [142] Acts on neural stem cells to induce their differentiation into astrocytes [143]
IL-8 *CXCL8* *GRO (the rat analogue)*	Produced by a wide variety of cells including monocytes, endothelial cells, T-cells, and macrophages [144] Binds to CXCR1 and CXCR2 receptors [52] Induces chemotaxis in neutrophils and granulocytes [52] Upregulated for at least 14 days after SCI and strongly correlates with the extent of injury [87, 49, 38, 14]
IL-10	Produced by monocytes, B-cells, dendritic cells, natural killer cells, and T-cells [145] Binds to IL-10R heterotetramer complex made of two IL-10R1 molecules and two IL-10R2 molecules [146] Downregulates several pro-inflammatory cytokines and inflammatory species [56] Provides trophic support to neurons through downregulation of pro-apoptotic factors and upregulation of anti-apoptotic factors [56]
IL-12 *IL-12ɑ* *P35*	Produced by dendritic cells, macrophages, monocytes, neutrophils, microglia cells, and B-cells [147] Binds to IL-12R, which consists of the IL-12Rβ1 and IL-12Rβ2 chains [148] IL-12(p70) expresses nitric oxide synthase and TNFα in microglia and PDMs [149]
IL-13	Produced by T-cells, dendritic cells, and activated Th2 cells [150] Binds either to IL-13Rɑ1 or IL-13Rɑ2 [53, 151] Involved in the production of transforming growth factor beta (TGF-β) [151]
IL-17ɑ *IL-17* *CTLA-8*	Produced by several types of cells including T-cells, dendritic cells, and macrophages [152] Binds to the A and C subunits of IL-17R [152] IL-17 knockout mice showed increased locomotor function after SCI suggesting a role in regulating secondary degeneration of neural tissue [153]
TNF-ɑ *TNF* *TNFSF2*	Produced by microglia, PDMs, astrocytes, oligodendrocytes, monocytes, and neurons [37, 39] Two active forms are transmembrane TNFɑ and soluble TNFɑ [154] Soluble TNFα is released by regulated cleavage of transmembrane TNFɑ by TNFɑ-converting enzyme (TACE/ADAM17) [154] Binds to TNFR1 and TNFR2 [154] Affects cell proliferation, differentiation, apoptosis, immunity, and inflammation [37] Recruits macrophages to injury site [37]
IFN-γ	Produced by γẟ T-cells and leads to the activation of macrophages [95, 155] Induces secretion of IL-10 from microglia and PDMs [155] Can act synergistically with GM-CSF to promote the production of cytokines [156]
GM-CSF *CSF2*	Produced by macrophages, mast cells, T-cells, fibroblasts, and endothelial cells in response to immune activation and cytokines that mediate inflammation [157] Causes differentiation of hematopoietic progenitor cells into granulocytes, macrophages, and dendritic cells [158] Can stimulate the proliferation of bone marrow stem cells and reduce leukocyte apoptosis, as well as cause microglia to proliferate and change their morphology [158] GM-CSF-induced activation of microglia may promote functional recovery and axonal regeneration by release of brain-derived neurotrophic factor or by phagocytosis of myelin debris [159, 160]
MCP-1 *CCL2*	Secreted by activated T-cells, astrocytes, microglia, and monocytes [161] Binds to CCR2 [106] Activates and recruits mononuclear phagocytes, T-cells, and B-cells [162]
MIP-1ɑ *CCL3*	Secreted by activated T-cells, astrocytes, microglia, and monocytes [161] Promotes chemotaxis of monocytes and T-cells [161] Expression induced by pro-inflammatory stimulators, such as LPS, TNFα and IL-1β, and neuronal injury [163] Can enhance the production of other pro-inflammatory cytokines via CCR1, CCR4, and CCR5 [163] Impacts CNS inflammation through regulation of macrophages and astrocytes [163]
RANTES *CCL5* *TCP228*	Produced by astrocytes and is primarily involved in promoting migration of M2 macrophages [51] Expression induced by IL-1 and macrophage migration inhibitory factor [51] Binds to CCR1, CCR3, and CCR5 [164] Associated with T-cell activation in SCI. Chronic T-cell activation subsequently contributes to neurodegeneration and inhibits repair of injured tissues [165] Induces the migration and recruitment of a wide variety of cells including T-cells, dendritic cells, natural killer cells, eosinophils, basophils, mast cells, and endothelial progenitor cells [51] May help amplify inflammatory response by facilitating the recruitment of inflammatory cells to the injury [51]
TGF-β1	Produced by lymphocytes, macrophages, and dendritic cells [166] Binds to TGF-βR1, TGF-βR2, and TGF-βR3 [167] Activates glial cells and phagocytes to form connective tissue and extracellular matrix [168] Influences the differentiation, proliferation, and state of activation of leukocytes [166] Known to suppress expression of MHC class II antigen [166]

Although the present review focuses primarily on the localized inflammatory response to SCI in rodent models, it is important to note that there is systemic inflammation occurring after SCI. Due to the inability to sample the spinal cord parenchyma of human patients after injury, many are investigating whether human blood serum and/or cerebrospinal fluid (CSF) cytokine samples may be used as biomarkers to provide insight into injury severity, thereby influencing treatment decisions [14–16]. Kwon et al. [14] found that at 24 h after injury, cytokines IL-6, IL-8, and MCP-1, along with structural proteins, such as GFAP, tau, and S100β, were present in significantly higher concentrations than corresponding serum samples in human patients. In the CSF of a rat SCI model 6 h after injury, TNFɑ, IL-2, IL-10, IL-17ɑ, and IFN-γ concentrations were significantly increased [17], and serum concentrations of TNFɑ, IL-1β, and IL-6 have been shown to remain elevated in rat throughout the first week following SCI [18–20]. This is similar to the local upregulation of these cytokines that occurs within the injured spinal tissue (Figs. 2 and 3). Interestingly, the upregulation of proinflammatory cytokines TNFɑ and IL-1β persists in the serum 28 days after SCI in a rat, while IL-4 is downregulated at 28 days and there is no significant change in IL-10 at this timepoint [21]. However, Ogurcov et al. [15] found a decreased concentration of both IL-1β and IL-10 in serum of human patients 14 days after SCI. Furthermore, they report the elevation of IL-4 and IFN-γ levels at this timepoint [15]. In general, analysis of cytokine in CSF and serum display similar patterns as the local response in injured spinal tissue (Figs. 2 and 3).

Locally, the primary trauma after SCI, injures the glial cells in the spinal cord and damages blood vessels causing ischemia. The death of nearby glial cells and the ischemia are the initial structural and cellular damage that triggers the secondary injury cascade. The primary cytokines that have been studied extensively within this cascade are listed in Table 1 in terms of the cells that secrete them, receptors they bind to, and their effects after SCI. Below, we discuss the timeline of this cascade in terms of cytokine/chemokine regulation and cellular infiltration.

### Within the first hour post-injury

Ischemia, oxidative damage, edema and glutamate excitotoxicity all start minutes after SCI and contribute to substantial secondary damage [22–25]. Cell permeabilization, pro-apoptotic signaling and ischemic injury due to the destruction of the microvascular supply cause additional cell dysfunction and death just minutes after SCI [26, 27]. There is also intracellular calcium dysregulation in both neurons and glia, resulting in the activation of calpains, which can cause mitochondrial dysfunction and cell death [25, 28, 29]. Microglia, the resident immune cells, quickly respond to the injury and their early response is protective [30–32]. However, microglia rapidly change to proinflammatory cells and release cytokines setting off a cascade of events that lead to an infiltration of peripheral immune cells [12]. The activated microglia retract their cytoplasmic processes and become indistinguishable in terms of morphology from the PDMs, which enter from damaged blood vessels [33–36]. Within 30-min post-injury, microglia and astrocytes begin expressing inflammatory cytokines IL-1β and TNFɑ mRNA [37]. Pineau and Lacroix showed that TNFɑ mRNA positive cells, including microglia, astrocytes, oligodendrocytes, and neurons, peaked just 1 h after mice sustained a spinal cord contusion [37].

### Cytokine signaling from 1-h up to 3-h post-injury

Using colocalization studies for mRNA after SCI in mice, Pineau and Lacroix showed that during the first few hours, proinflammatory cytokines IL-1β, TNFɑ, and IL-6 were being synthesized by microglia/macrophages, astrocytes, and neurons [37]. Using various techniques to measure TNFα in these early hours after SCI, five other studies all observed significant increases in TNFɑ levels (Fig. 2B) [38–42]. There were also five studies that measured IL-1β using various techniques in these early hours after SCI and three of these studies observed a significant increase in IL-1β (Fig. 2A) [38, 40, 41]. However, two experiments utilizing enzyme-linked immunosorbent assays (ELISAs) to measure IL-1β did not observe an increase at this early timepoint [42, 43], which may be the result of the discrepancy in time needed to synthesize the full protein and the time needed for it to be proteolytically processed to its active form by caspase 1 [44]. There were very similar trends in the early IL-6 upregulation to that of IL-1β (Fig. 2C) [38, 40–42]. The proinflammatory cytokines TNFɑ, IL-1β, and IL-6 are presumed to be the predominant players early in the injury timeline. Thus, these three cytokine levels were the most frequently analyzed in these early hours after SCI (Fig. 2).

### Cytokine signaling and cellular reactions from 3-h up to 6-h post-injury

At this early stage of inflammation, the majority of the literature agrees that the primary inflammatory cytokines, TNFɑ, IL-1β, and IL-6, are all significantly upregulated (Fig. 2) [37, 38, 40, 45–48]. Pineau and Lacroix observed that at 3 h after SCI in mice, the number of cells expressing the TNFɑ mRNA transcript at the lesion epicenter was significantly upregulated compared to uninjured controls. However, the number of cells had decreased by 66% compared with the 1-h timepoint [37]. Two research groups did not observe a significant increase in TNFɑ protein expression at 4 h after a rat contusion, which suggests a return to baseline after a rapid onset of TNFɑ [38, 40]. Although the upregulation of IL-1β and IL-6 lags slightly behind that of TNFɑ and IL-1ɑ, most of the literature agrees that IL-1β and IL-6 are significantly upregulated within this 3–6-h time window [37, 38, 40, 42, 45–47]. The expression of growth-related oncogene (GRO), the rat analogue of IL-8, is also significantly upregulated at 4-h post-injury [38, 45, 49, 50]. The cytokine regulated upon activation, normal T-cell expressed and presumably secreted (RANTES), which induces migration and recruitment of T-cells, eosinophils, basophils, and leukocytes, has been shown to be significantly upregulated 4-h post-injury, although it is not as prevalent in the literature [38, 47, 51]. This early upregulation in inflammatory cytokines is produced predominantly by microglia and astrocytes and leads to the recruitment of peripheral immune cells.

Although anti-inflammatory cytokines are capable of reducing proinflammatory cytokines, they are generally present at low levels or absent after SCI. IL-4 and IL-13 are related anti-inflammatory cytokines that have been shown to induce alternative macrophage activation [52–55], and IL-10 is an anti-inflammatory cytokine that has been shown to downregulate pro-inflammatory cytokines [56, 57]. Four hours after SCI in mice, IL-10 was significantly upregulated [47], yet, at this same time post-injury in rats IL-10 was not upregulated [38, 46]. In contrast, IL-13 was upregulated in rats [38, 48] but not in mice [47]. A number of researchers have shown that there are differences in the adaptive immune system between mice and rats as well as between different strains of mice, which may explain the discrepancies in anti-inflammatory cytokine regulation [12, 58–60].

**Fig. 4:**
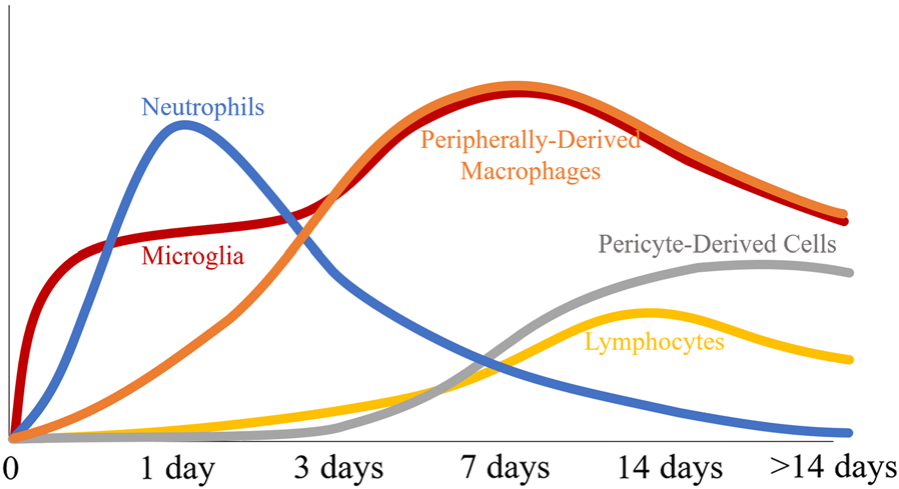
Primary injury after SCI causes cell membrane disruption and rupture of blood vessels leading to secondary injury with extensive upregulation of cytokines/chemokines and infiltration of immune cells. Microglia, the resident macrophages, are early responders and become indistinguishable in terms of morphology from peripherally derived macrophages. In rats, neutrophils peak early in the injury at around 24 h and gradually decrease over the next 7–10 days, while lymphocytes peak at lower levels and at a much later time. Pericytes also infiltrate later and interact with microglia around the edges of the injury and this entire injury site is encased by activated astrocytes

The majority of the literature shows that in this 3–6-h post-injury period there is an upregulation in inflammatory cytokines, which are produced largely by local microglia and astrocytes, leading to further recruitment and proliferation of microglia and astrocytes and the recruitment of peripheral immune cells (Fig. 4). Neutrophils begin to appear 4–6 h after SCI and produce oxidative and proteolytic enzymes to sterilize and prepare the area for repair [61, 62]. However, overwhelming numbers of neutrophils result in tissue damage [61].

Lipid peroxidation, the process by which free oxygen radicals react with the polyunsaturated fatty acids of membrane lipids, leads to 4-hydroxynonenal (4-HNE) formation and disrupts cell membrane fluidity, metabolic processes, and ion transport systems [63, 64]. 4-HNE itself is neurotoxic and can bind to cellular proteins and damage their structural and functional integrity [65]. Xiong et al. [65] also observed that byproduct formation such as 4-HNE increases as soon as 3-h post-injury and remains high for 2 weeks.

### Cytokine signaling and cellular reactions from 6-h up to 12-h post-injury

Almost all of the literature shows that the upregulation in proinflammatory cytokines TNFɑ, IL-1β, and IL-6 continues through the 6–12-h timeframe post-injury (Figs. 2 and 3) [17, 37–39, 41, 42, 66–69]. There is also a significant increase in monocyte chemoattractant protein 1 (MCP-1) [17], macrophage inflammatory protein 1-alpha (MIP-1ɑ) [17], RANTES [17], GRO [17, 50], and C–X–C motif chemokine ligand 1 (CXCL1) [69]. The majority of the literature shows that IL-4, IL-10, and IL-13 remain at baseline levels (Fig. 2G–I) [17, 38, 66, 70]. Ultimately, this inflammatory environment continues the recruitment and proliferation of microglia and astrocytes and the recruitment of peripheral immune cells. Apoptosis from this exacerbated inflammation process after SCI peaks near 8 h in neurons and around 24 h in glial cells [71, 72].

During these early hours after injury, polymorphonuclear leukocytes are the predominant infiltrating cells [73], and over-activation of these cells continue to cause tissue destruction through the release of significant amounts of neurotoxins including reactive oxygen species (ROS), reactive nitrogen species (RNS), chemokines, and enzymes [4, 61, 74, 75]. Popovich et al. [32] also showed that lymphocytes begin to accumulate around blood vessels in gray matter as early as 6 h after SCI in rats.

After SCI, activated microglia and PDMs have the capability to take on different functional phenotypes [33, 57, 63, 76–80]. Resolution-phase macrophages are enriched with major histocompatibility complex II (MHC) expression [81], express anti-inflammatory cytokines TGF-β1 and IL-10 [81, 82], and have elevated expression of key enzymes involved in synthesizing pro-resolving mediators that actively turn off inflammation [83]. In vivo, macrophages in general are not fully inflammatory (M1) or anti-inflammatory (M2), but rather exist on a continuum in which their roles and phenotype are dictated by the extracellular environment [57]. As early as 6 h after SCI in mice, Kigerl et al. [12] observed antigen-presenting MHCII cells in the dorsal roots, meninges, and within a subset of perivascular spaces.

### Cytokine signaling and cellular reactions from 12-h up to 24-h post-injury

The majority of the literature continues to show elevated levels of TNFɑ, IL-1β, and IL-6 (Fig. 2A–C). There are also increased levels of GRO [50, 84], IL-7 [48], and chemokines MCP-1 [7, 85], and MIP-1α [84]. IL-4, IL-10, IL-13 and TGF-β1 all remain at baseline levels (Fig. 1G–I) [41, 70, 85]. Although IL-4 was not upregulated, Francos-Quijorna et al. [70] observed an upregulation of IL-4Rɑ on microglia and macrophages.

Cells expressing IL-1β and cells expressing IL-6 both peaked at 12-h post-injury [37]. Infiltrating leukocytes also produce IL-1β, TNFɑ, IL-6, and LIF 12-h post-injury [37]. The infiltrating neutrophils, which phagocyte and clear debris, secrete proteases, elastase, myeloperoxidase, and release reactive oxygen species, peak at 24-h post-injury [86]. The byproduct formation of 4-HNE also peaked at 24 h and remained elevated for 2 weeks [65].

### Cytokine signaling and cellular reactions from 24 h up to 7-day post-injury

Pineau and Lacroix’s colocalization studies showed that TNFɑ mRNA levels 2-day post-injury had returned to the levels detected in mice that had received laminectomy only [37]. The number of IL-1β positive cells continued to decrease at 2-, 4- and 7-day post-injury, and from 24 h up to 4 days, the average number of cells expressing the IL-6 transcript progressively decreased to become almost nonexistent at 7 days [37]. Despite the decrease observed in the number of cells expressing mRNA, several researchers observed levels that were significantly higher than their uninjured controls at 1 day (TNFɑ 77.8%, IL-1β 79.2%, IL-6 88.2%), 3 days (TNFɑ 80.1%, IL-1β 68.8%, IL-6 73.7%), and 7 days (TNFɑ 64.3%, IL-1β 41.7%, IL-6 23.1%) after SCI (Figs. 1 and 2). These trends were similar in the literature for both rats and mice after SCI. GRO was also significantly elevated on day 1 [50, 84], day 3 [84, 87], and day 7 [87].

There is significant upregulation of chemokines throughout this first week recruiting monocytes, T-cells, and dendritic cells to the injury site. MIP-1α is upregulated on day 1 [84], day 3 [84, 87], and day 7 [87]. When tested, CXCL1 expression is significantly increased on day 3 [69, 88, 89] and day 7 [86] and RANTES is significantly upregulated on day 3 [47, 88–90]. Previous studies have shown that MCP-1 mRNA is increased in mice 1-day post-injury and returns to baseline before 7-day post-injury [91, 92]. However, here there are some conflicting results 7-day post-injury. Although studies looking at mRNA MCP-1α levels 7-day post-injury observed a significant increase in mice [93] and rats [85], a study using multiplex assay 7-day post-injury in rats observed a significant decrease in MCP-1α levels (Fig. 2F) [87]. MIF and COX2 were inducibly expressed after SCI in rats and peak levels were observed on day 4 [94].

Some cytokines displayed contrasting behaviors between rat and mouse models during the 24-h to 7-day period. In mice, IFN-γ levels were significantly increased 1 day [48, 95], 3 days [48], and 7 days [48] after injury, but IFN-γ levels in rats were significantly decreased at 3 days [87] and 7 days [87] following SCI (Fig. 2E). Levels of IL-2 and IL-5 were significantly decreased in rats on day 3 and day 7 post-injury [87], where in mice there was no change in IL-2 levels [88], and a significant increase in IL-5 levels 3-day post-injury [89]. IL-13 was also significantly decreased 3 days and 7 days after SCI in rats [87]. However, in mice IL-13 was significantly upregulated 1 day and 3 days after SCI [48, 90] and returned to baseline levels 7-day post-injury (Fig. 1I) [48]. Similarly, IL-4 levels were not changed 1 day [38, 66], 3 days [7, 66, 87], and 7 days [7, 87] after SCI in rats, but were significantly increased 1 day, 3 days, and 7 days post-injury in mice (Fig. 2H) [48].

The majority of researchers did not observe a significant increase in the anti-inflammatory cytokine IL-10 until 3-day post-injury, which remained elevated until 7 days after SCI (Fig. 2G). There were also several researchers that did not observe changes in IL-10 levels after SCI in both mice [90] and rats [38, 66]. TGF-β1 was significantly increased 3-day [41] and 7-day post-injury [41, 96].

Using flow cytometry after SCI in rats, Beck et al. [97] demonstrated a time-dependent multiphasic response of cellular inflammation, where the initial phases of cellular inflammation were composed of an early peak of neutrophils 1-day post-injury, followed by a peak of macrophages/microglia 7-day post-injury, and T-cells peaking at 9-day post-injury. This timeline of cellular inflammation in rats with SCI is in agreement with other literature for neutrophils [61, 62], macrophages [62, 98, 99], and lymphocytes [98] (Fig. 4). After SCI in mice, neutrophils enter the injured spinal cord within 6 h, but surprisingly do not reach peak numbers until 14-day post-injury and persist for up to 6-week post-injury [12].

**Fig. 5:**
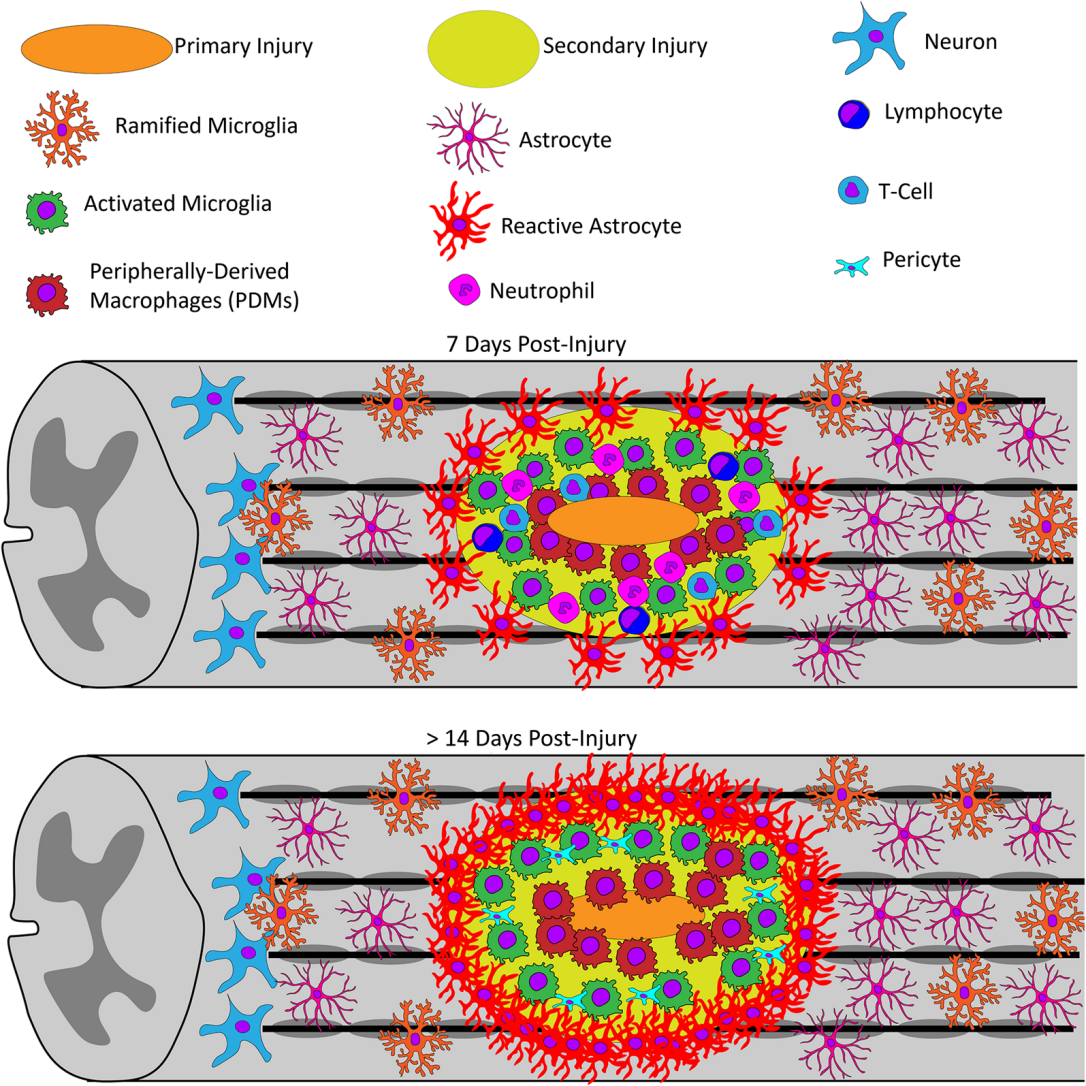
Diagram displaying cellular activation/infiltration after rat SCI. After the primary insult, there is a much larger secondary injury with extensive infiltration of immune cells. Neutrophil infiltration peaks 24-h post-injury and decreases over the next week. Infiltrating lymphocytes accumulate around blood vessels in gray matter as early as 6 h and T-cell specific lymphocytes peak around 9-day post-injury. Microglia are activated, retracting their cytoplasmic processes and becoming indistinguishable in terms of morphology from the infiltrating PDMs. The astrocytes also become reactive and retract the cytoplasmic processes and migrate to the lesion. Although initially the astrocytes aid in tissue repair, they eventually become scar-forming astrocytes and begin to wall off the ensuing inflammation. The final glial scar is compartmentalized with infiltrating immune cells in the center, microglia interacting with pericytes around the edges, and astrocytes encapsulating the entire tissue containing the inflammatory cells

Although axons begin to retract within 2-day post-injury due to the initial trauma, there is a later phase of axon retraction caused by activated macrophages [63, 100–104]. Bisicchia et al. [105] demonstrated that autophagosomes mediating the initiation of autophagy accumulate in axotomized neurons 1 day after SCI and remain high from 3 to 5 days before falling slightly 7-day post-injury. By 5-day post-injury, activated microglia and PDMs are the predominant inflammatory cells, where phagocytic macrophages are mostly located in areas of necrosis and microglia are at the margins. Lendate et al. [99] showed that microglia are highly dynamic, proliferate extensively the first week after SCI, and form a dense cellular interface at the border of the lesion between reactive astrocytes and infiltrating PDMs (Figs. 4 and 5).

### Cytokine signaling and cellular reactions from 7-day up to 14-day post-injury

A second surge was observed in cells expressing TNFɑ and IL-1β 14 days after SCI in mice [37]. This second upregulation of TNFɑ and IL-1β appears to only occur in mice [37, 48, 106], and not rats [87, 107] (Fig. 2A, B). IL-6 appears to be returning to baseline in both rats and mice by 14-day post-injury [37, 87, 90, 93, 107] (Fig. 1C). Mukhumedshina et al. [87] measured cytokine levels 14 days after SCI in a rat and observed a significant increase in IL-1ɑ, GRO, IL-2, and MIP-1ɑ, and a significant decrease in levels of IL-13, IL-2, IL-5, IL-18, IL-17ɑ, and GM-CSF. IL-1ɑ, IL-4, IL-12, IL-15, RANTES, MIP-1ɑ, MCP-1, and IL-7 are significantly upregulated in mice 14-day post-injury [48, 90, 93]. Beyond day 14 post-injury, there is less data measuring cytokine levels for comparison (Fig. 2).

Although there is proliferation of oligodendrocyte progenitor cells and maturation over the first 2-week post-injury, the end result is still improper remyelination [63, 108, 109]. The response of microglia and PDMs are similar after SCI in mice, rats, and humans, where peak numbers are reached around 7-day post-injury and remain in the injury site for months after injury [57, 97–99, 110, 111]. In contrast, lymphocyte infiltration is much larger in mice than in rats or humans and peaks at a much later timepoint (14-day post-injury) in mice [98, 110, 111]. Also unique to mice is the infiltration of fibroblast-like cells and the formation of fibrotic tissue matrix [110]. It is suggested that the infiltration of fibroblast-like cells coupled with the larger T-cell infiltration may be responsible for minimizing the development of cysts in mice [110, 111].

### Cytokine signaling and cellular reactions greater than 14-day post-injury

Astrocytes are the predominant subtype of glial cells in the central nervous system that work to maintain neurons as well as the blood spinal cord barrier [112]. Although initially reactive astrocytes migrate to the lesion and aid in the tissue repair process after SCI, they eventually become scar-forming astrocytes and form a glial scar around the lesion [112]. This thick layer of astrocytes surrounding the injury site is characterized by cellular hypertrophy, process extension, and the increased expression of intermediate filaments, such as glial fibrillary acidic protein (GFAP) (Fig. 5) [112].

T-cells are responsible for cell mediated adaptive immunity [113]. Infiltration of T-cells into the injured spinal cord occurs at different times depending on the species and strain of animal [110, 111]. After SCI, T-cell proliferation and cytokine production is further induced by chemokines CXCL10 and RANTES [113–116]. It is still a controversial topic on whether T-cells cause secondary degeneration or mediate wound repair after SCI [111, 113, 117, 118]. Gonzalez et al. [119] neutralized the chemoattractant CXCL10 and showed that it limited T-cell infiltration and improved tissue preservation and functional recovery, suggesting a negative role for T-cells. After SCI in rats, Wu et al. [120] observed that the dominant T-cells were cytotoxic T-cells (> 90%) with small numbers of regulatory T-cells (< 10%), which again suggests more of a destructive role in regard to tissue recovery. Furthermore, Lui et al. [121] showed that T-cell-derived perforin destroys the blood spinal cord barrier after SCI, resulting in an infiltration of inflammatory cytokines aggravating secondary injury. After SCI in mice, T-cell infiltration peaks around 14 days, decreases for approximately 2 weeks, then increases again over the next few weeks to reach amounts similar to the level at 14-day post-injury, and are still detected for at least 42-day post-injury [12, 120, 122–125].

After SCI, pericytes, the main source of the scar connective tissue, enter the lesion area with blood vessel sprouts and form the stromal component of the scar tissue [126]. Pericytes can also be activated by trace amines to locally constrict the vasculature [127]. Thus, pericytes regulate the capillary tone and blood flow in the spinal cord after injury. After SCI in mice, the number of pericyte-derived cells peaked at 2 weeks, which was shown to be more than a 25-fold increase, and then decreased after 4 months before leveling off and remaining for at least 7 months [126]. The final glial scar is compartmentalized with infiltrating immune cells in the center, microglia interacting with pericytes around the edges, and astrocytes surrounding the periphery (Fig. 5) [99, 126].

## Conclusions

Over the past few decades, there have been a plethora of researchers studying the complex inflammatory process that follows SCI. This research has helped to define the critical timeline of cell infiltration and cytokine profiles that occur after SCI. The majority of this work has been performed using rodent models. Although there are many similarities in both rats and mice after SCI, differences exist in terms of neutrophil infiltration, lymphocyte infiltration, fibrotic scarring, and an absence of cysts in mice. Overall, these rodent models have served well in defining the cytokines, chemokines, and reactive species involved after SCI.

To date, there is no successful treatment for SCI patients to either help retain function after the injury, or regain function through regeneration after the injury. Many scientists are in agreement that adapting/minimizing the secondary injury that occurs after SCI would be a premiere target for treating SCI patients. Thus, research needs to continue in this area to discover new mechanisms to modulate inflammation after SCI.

## Data Availability

The data sets used and analyzed during the current study are included within the article and its additional files. All material used in this review are from publicly available articles.

## Abbreviations

4-HNE: 4-Hydroxynonenal
CASP1/ICE: Caspase-1/interleukin-1 converting enzyme
CXCL1: C–X–C Motif Chemokine Ligand 1
CNS: Central nervous system
CSF: Cerebrospinal fluid
COX: Cyclooxygenase
ELISA: Enzyme-linked immunosorbent assay
GFAP: Glial fibrillary acidic protein
GM-CSF: Granulocyte–macrophage colony-stimulating factor
GRO: Growth-related oncogene
IFN-γ: Interferon gamma
IL: Interleukin
MCP-1: Monocyte chemoattractant protein 1
MeSH: Medical Subject Headings
MHC: Major histocompatibility complex
MIF: Macrophage migration inhibitory factor
MIP-1ɑ: Macrophage inflammatory protein 1-alpha
NK: Natural killer
PDMs: Peripherally derived macrophages
RANTES: Regulated upon activation, normal T-cell expressed and presumably secreted
RNS: Reactive nitrogen species
ROS: Reactive oxygen species
SCI: Spinal cord injury
TACE/ADAM17: Tumor necrosis factor alpha converting enzyme
TGF-β: Transforming growth factor beta
TNFɑ: Tumor necrosis factor alpha
